# Reliability of mechanical properties of the plantar flexor muscle tendon unit with consideration to joint angle and sex

**DOI:** 10.1371/journal.pone.0287431

**Published:** 2023-06-23

**Authors:** Evan D. Crotty, Laura-Anne M. Furlong, Andrew J. Harrison

**Affiliations:** Sport and Human Performance Research Centre, University of Limerick, Limerick, Ireland; Università Telematica degli Studi IUL, ITALY

## Abstract

The reliability of mechanical measures can be impacted by the protocol used, including factors such as joint angle and the sex of participants. This study aimed to determine the inter-day reliability of plantar flexor mechanical measures across ankle joint angles and contraction types and consider potential sex-specific effects. 14 physically-active individuals participated in two identical measurement sessions involving involuntary and voluntary plantar flexor contractions, at three ankle angles (10° plantarflexion (PF), 0° (anatomical zero (AZ)), and 10° dorsiflexion (DF)), while torque and surface EMG were recorded. The reliability of mechanical parameters of maximal voluntary torque (MVT), rate of torque development (RTD), electromechanical delay, and tendon stiffness were assessed using absolute and relative reliability measures. MVT measures were reliable across ankle angles. RTD measures showed good group level reliability and moderate reliability for an individual during the early phase of contraction across ankle angles. Explosive voluntary torque measures tended to be less reliable from 50 ms onward, with varied reliability across angles for late-phase RTD. Tendon stiffness demonstrated the best reliability at the DF angle. Sex-based differences in the reliability of tendon measures found that females had significantly different initial tendon length between testing sessions. Despite this, tendon excursion, force, and stiffness measures demonstrated similar reliability compared to males. Ankle angle changes influence the reliability of plantar flexor mechanical measurements across contraction types, particularly for voluntary contractions. These results highlight the importance of establishing potential protocol effects on measurement reliability prior to quantifying plantar flexor mechanical measures.

## Introduction

Establishing the reliability of measurement is essential, so that differences observed over time or between participants may be attributed to physiological changes, and not random variation. The triceps surae muscle group is commonly examined in neuromuscular and mechanical research due to its importance in locomotion [1, 2]. Mechanical performance of a muscle-tendon unit is determined by multiple factors including structural, morphological, and mechanical characteristics [3, 4]. Plantar flexor muscle torque is a primary determinant of mechanical performance, its importance exemplified by its contribution to accelerated sprinting [5], a key aspect of multiple sports. Consequently, the torque producing capabilities of this muscle group are commonly examined. In terms of torque production, it is influenced by factors such as muscle size, and architecture as well as sex and muscle-tendon unit length [6–8].

Plantar flexor muscle torque is commonly examined through outcome measures of maximal muscle torque, and the rate of torque development (RTD). Maximal muscle strength is an important determinant of mechanical performance. However the speed of most sporting movements restricts the production of peak torque [3, 4]. RTD is a more representative descriptor of functional performance and is defined as the rate of rise in contractile torque from the onset of contraction [9]. The reliability of plantar flexor torque measures has been previously established. Plantar flexor maximal strength has demonstrated acceptable reliability [1, 10]. Additionally, plantar flexor RTD, examined both voluntary and involuntary, has generally demonstrated moderate-to-high levels of intra-day [11] and inter-day [1] measurement reliability. Quantifying these primary determinants of plantar flexor performance is important in mechanical assessment, however establishing the underpinning mechanisms to these determinants is critical to truly understand muscle performance. Despite this, the reliability of mechanisms underpinning these determinants of plantar flexor performance are not well established.

The reliability of torque output measures can be influenced by various factors. Most notably, factors such as protocol, participants, number of trials, analysis method, and testing devices are reported to impact reliability [12]. Examination of measurement reliability research suggests that modifying joint angles, inducing a change in muscle-tendon unit length, could influence the reliability of mechanical performance measures [10, 13]. A comparison of plantar flexor RTD reliability across studies demonstrates varying reliability, potentially due to different ankle angles the researchers employed [11, 14]. Additionally, maximal muscle strength demonstrated varying reliability across different knee joint angles [10]. However, the reliability of plantar flexor determinants and mechanisms across ankle angles is not well established. Female hormones, namely estrogen, progesterone, and relaxin have a significant impact on connective tissue [15–17], thus fluctuations in these hormones across a menstrual cycle may influence the reliability of mechanical measures. While muscle is responsive to estrogen fluctuations, there is evidence to suggest the tendon is more influenced by estrogen levels [15–17]. Thus, establishing reliability of tendon measures in the respective sexes is important for future studies examining tendon function in these participant groups.

The reliability of mechanical measures can be impacted by changes in muscle-tendon unit length [10], however the influence of changes in ankle joint angle on mechanical measures of plantar flexor performance and their determinants has not been investigated. Identifying the joint angle that provides the best reliability when assessing mechanical outcome measures and their determinants needs to be established. Many studies examine reliability on consecutive days or within sessions (intra-session reliability). However, this study focused on reliability over time, as this may be practically relevant for intervention studies conducted over several weeks. Thus, the aim of this study was to determine the inter-day reliability of plantar flexor outcome measurements of maximal voluntary torque (MVT), and RTD (voluntary and involuntary), alongside contributing mechanisms of electromechanical delay (EMD; voluntary and involuntary), tendon stiffness, and tendon measures (force, elongation, strain, and resting tendon length) across three different ankle joint angles: 10° plantarflexion (PF), 0° (anatomical zero (AZ)), and 10° dorsiflexion (DF). Additionally, the study examines the sex-specific reliability of tendon measures for males and females and any sex-related differences in inter-day reliability.

## Materials and methods

### Participants

A convenient sample of 14 participants (7 males, 7 females; age: 25.6 ± 1.8 years, height: 1.70 ± 0.07 cm, mass: 70.1 ± 6.9 kg,) of similar moderate-to-high levels of habitual physical activity were recruited for the study (IPAQ short format, http://www.ipaq.ki.se/index.html; [18]). Previous research examining the influence of joint position on the reliability of mechanical variables [2, 13] used similar sample sizes to the sample size in the current study. To control for the potential of the menstrual cycle phase influencing the mechanical performance of the plantar flexors, female participants were required to have been taking the combined monophasic oral contraceptive pill (OVREENA n = 5; Microlite n = 2) for ≥ 6 months and were only tested between days 7 and 21 of pill consumption to minimize fluctuations in endogenous gonadal hormones [19]. Participants were excluded if they had experienced a lower-leg injury in the previous six months. All testing procedures were approved by the University of Limerick Education and Health Sciences Research Ethics committee and all participants provided written informed consent.

### Protocol

**Fig 1:**
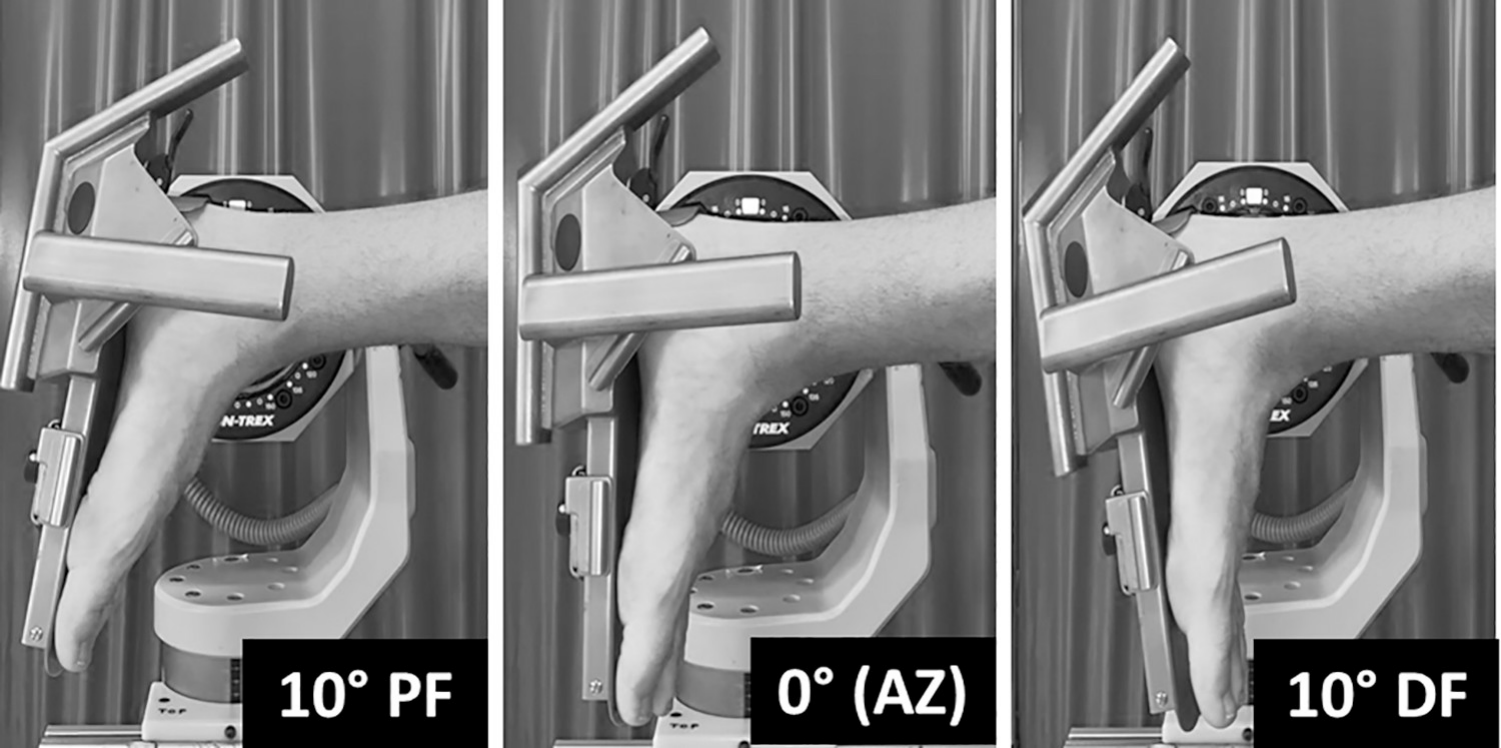
Dynamometer setup and ankle joint testing angles.

The mechanical function of the right plantar flexor muscle group was assessed on two occasions separated by three to seven days to limit the residual effects of testing between bouts [20]. This time frame between repeated assessments provides a reasonable compromise between recollection bias and unwanted clinical change in the participants. The inter-session reliability of mechanical variables were measured across three different ankle joint angles (10° PF, 0° (AZ), 10° DF; Fig 1). AZ was defined as the neutral angle between the foot and shank (90°). Participants visited the laboratory for 75–120 min on three occasions, to complete a familiarization and two testing sessions. Participants were requested to refrain from strenuous physical activity 24-h before the testing session and avoid caffeine consumption the day of the testing session. All contractions at a given joint angle were completed in one block, with joint angle order randomized. The order of measurements was: 1) electrically evoked twitch contractions, 2) explosive voluntary contractions, and 3) maximal voluntary contractions.

### Data recordings

#### Electrically evoked twitch contractions

A constant current, variable voltage stimulator (DS7AH, Digitimer Ltd, Hertfordshire, UK) electrically stimulated the posterior tibial nerve with square wave pulses (0.1 ms) to evoke a twitch contraction. The cathode was attached to the skin over the tibial nerve in the popliteal fossa and the anode was attached to the skin on the distal aspect of the thigh, proximal to the patella. A series of discrete single pulses were delivered at incremental current intensities until a plateau in muscle twitch force and M-waves was reached. The stimulus intensity was then increased by a further 20% to ensure supramaximal stimulation. Three supramaximal stimuli were delivered at each joint angle, separated by 12 s rest between stimuli and 2 minutes rest between ankle joint angles. The stimulator output was simultaneously recorded on LabChart 8 software (AD Instruments, Sydney, Australia) using an analog-to-digital converter.

#### Explosive voluntary contractions

Explosive isometric voluntary contractions of the plantar flexors were performed at each joint angle following a standardized warm-up. Participants performed five contractions at each joint angle, with 20 s rest separating each contraction and 2 minutes between joint angles. They were instructed to plantar flex their ankle ‘as fast and hard as possible, with an emphasis on fast’ for 1 s upon hearing an electronic auditory signal. At each joint angle, the three contractions with the largest peak slope and no countermovement were used for calculating mean values for each participant.

#### Maximal voluntary contractions–maximal voluntary torque measurement

Participants performed three maximal isometric voluntary contractions of the plantar flexors at each joint angle, with two minutes rest between each contraction and five minutes rest between sets. Participants were secured lying prone in a calibrated dynamometer (Con-trex, Dubendorf, Switzerland). The dynamometer and ankle joint (at neutral angle) axes of rotation were aligned, with the knee joint fixed at 180° in full extension. The ankle was securely fastened with two straps, one 2 cm proximal to the medial malleolus and one 3 cm proximal to the head of the first metatarsophalangeal joint. Participants were instructed to plantarflex their ankle ‘as hard as possible without any concern for the rate of force development’. They were further instructed to build to maximum plantar flexion over 3 s and to maintain this for a further 1–2 s. Real-time torque biofeedback on a computer monitor was provided. Additionally, intense verbal encouragement from a standardized script was provided during the contractions. Heel lift was monitored using an electrogoniometer, with trials repeated if ankle angle changed >2° from the testing angle during measurement. The torque signal from the dynamometer was amplified (x1000) and sampled at 2000 Hz using an external analogue to digital converter (PowerLab 4/20; AD Instruments, Sydney, Australia) interfaced with a computer running LabChart 8 software (AD Instruments, Sydney, Australia).

#### Maximal voluntary contractions—tendon stiffness measurement

During the maximal voluntary contraction, muscle-tendon unit stiffness was examined. Muscle-tendon unit stiffness was evaluated following previously established methods [21, 22] and examined during maximal voluntary contractions. Muscle-tendon unit elongation was examined using a 7.5 MHz 128-element linear ultrasound probe (LV7.5/60/128Z-2; LS128 CEXT-1Z; Telemed, Vilnius, Lithuania), recording at 40 Hz. The ultrasound probe was positioned on the skin and the probe head was aligned parallel to the muscle fascicles. To measure the elongation of the tendon, the probe was positioned at the most distal aspect of the MG, at the junction with the Achilles tendon. The distal end of the tendon was represented by a marker placed on the calcaneus to estimate the insertion of the tendon on the calcaneal tuberosity. An echogenic marker was positioned between the ultrasound probe and the skin to measure any probe motion relative to the skin. An electrogoniometer (Biometrics, Gometz-le-Châtel, France) was attached to the lateral surface of the participant’s lower leg (superior to the lateral malleolus) and the lateral surface of the fifth metatarsal to measure changes in ankle joint angle during the maximal contractions. Prior to the maximal voluntary contractions, the ankle of the participant was passively flexed and extended from 20° PF to 10° DF at 5°/s with synchronous ultrasound imaging of the MG aponeurosis, ankle joint angle, and torque. This passive assessment was performed to determine aponeurosis displacement resulting from passive ankle joint PF, and thus correct for this displacement that inevitably occurs during the maximal voluntary contractions. To estimate the resting length of the Achilles tendon, the length of the linear distance between the insertion of the tendon on the calcaneal tuberosity and the MG muscle-tendon junction was measured with the knee at 180° and the ankle at 20° PF [23]. During the measurement of resting tendon length, participants were instructed to relax their leg muscles.

#### Electromyography

EMG signals were recorded from the soleus, gastrocnemius lateralis, and gastrocnemius medialis using a DELSYS Trigno EMG system (Delsys, Boston, MA). Surface EMG for the Non-Invasive Assessment of Muscles (SENIAM 2008) guidelines guided the placements of the electrodes. Ultrasonography was used to optimize electrode sensor location based on the location of the midportion of the muscle belly and in line with the muscle fascicles (and hence muscle fibers). The EMG signals were amplified (x100; differential amplified, 20–450 Hz), sampled at 2000 Hz, and interfaced with LabChart 8 software with a wireless connection software.

### Data processing

Each participant had 3 trials for each contraction (electrically evoked twitch contractions, explosive contractions, maximal voluntary contractions) and at each joint angle (10° PF, 0° (AZ), 10° DF) included for analysis (27 trials in total per participant across contraction types and angles), with relevant variables extracted from each of these trials. All data treatment were performed in MATLAB (R2019a, MathWorks, 205 Massachusetts, USA) using a custom-written script.

#### Electromyography

During offline analysis, EMG data were full-wave rectified, band-pass filtered between 10 and 400 Hz with a fourth-order zero-lag Butterworth filter and conditioned with the Teager-Kaiser energy operator (TKEO) prior to analysis. The inclusion of the TKEO as a conditioning step has been shown to improve the reliability of manual visual detection [24].

#### Electrically evoked twitch contractions

Involuntary EMD was calculated as the time difference between the M-wave onset of the superficial plantar flexors and torque onset. M-wave onset and torque onset were identified manually as a positive or negative deflection away from the baseline. Involuntary rate of torque development (RTDI) in two 50 ms time windows (0–50, 50–100 ms) from torque onset was determined. RTDI was measured as absolute values and relative to MVT at the specific joint angle. Involuntary EMD, and RTDI measures extracted from each of the three analyzed contractions at each joint angle were averaged to provide an average angle value for each participant.

#### Explosive voluntary contractions

For each explosive contraction, voluntary EMD was calculated as the time difference between EMG onset and torque onset for each of the superficial plantar flexor muscles. Explosive torque was measured at 25, 50, 75, 100, 125, and 150 ms from torque onset and reported in absolute (N.m) and relative terms (%MVT). Relative explosive torque measures were normalized to the MVT at the specific joint angle. This method of normalization allows the comparison of an individual’s ability to utilize available MVT at a particular angle [25]. Voluntary explosive rate of torque development (RTDV) was quantified for three 50-ms time windows (0–50, 50–100, and 100–150 ms) from torque onset. RTDV was determined in absolute terms and normalized to MVT. Explosive EMD, premotor time, and RTDV measures extracted from each of the three analyzed contractions at each joint angle were averaged to provide an average angle value for each participant.

#### Maximal voluntary contractions

MVT was defined for each joint angle individually as the greatest torque during any of the maximal voluntary contractions at that particular joint angle. For tendon stiffness, the longitudinal displacement of the myotendinous junction of the MG was considered to represent the elongation of the Achilles tendon [26] and was tracked manually frame-by-frame using ImageJ software (ImageJ, National Institutes of Health, Bethesda, MD, USA). Tendon force was estimated by dividing the plantar flexor torque by the tendon moment arm. The moment arm of the Achilles tendon was estimated at rest for each test angle, by using a tape measure to measure the perpendicular distance from the medial malleolus to the line of action of the Achilles tendon and of the lateral malleolus to the line of action of the Achilles tendon. The mean of these measures was calculated to provide an estimate of the moment arm at each ankle joint angle. Absolute tendon stiffness (N/mm) was calculated as the slope of the Achilles tendon force-elongation relationship. Aponeurosis strain (*ε*) was calculated as the ratio of elongation to the initial resting length between 10–90% of maximal calculated tendon force during the maximal voluntary contraction. Absolute stiffness of the tendon was calculated as the slope of the calculated tendon force versus tendon elongation between 10% and 90% of the maximal tendon force. Angle-specific peak strain was quantified by normalizing elongation to initial tendon length at the test angle. Relative peak strain was quantified by normalizing elongation to resting tendon length at 20° PF [23]. Tendon stiffness measures were calculated using a custom-written MATLAB interface. Due to technical issues, ultrasound images were unclear for one participant, so muscle-tendon unit stiffness was not calculated for this individual.

#### EMG and torque onset detection

An individual with expertise in EMG and torque analysis manually determined the onset of all EMG and torque data traces across contractions. A custom-written script in MATLAB displayed individual EMG and torque traces on the computer monitor enabling onset detection to the nearest millisecond. For visual identification of onset, the criteria used by Tillin et al [3] was employed. Initially, EMG signals were viewed with a constant y-axis scale of 10 mV and an x-axis scale of 500 ms. Torque signals were viewed with a constant y-axis scale of 1 N and an x-axis scale of 500 ms. These scales enabled clear visualization of the pattern of noise and enabled signal onset detection. Signal onset was determined as the last peak or trough before the signal deflected away from baseline noise. Once signal onset was detected, the signals were viewed at a higher resolution (y-axis scale of 6 mV and 0.5 N for EMG and torque respectively, with an x-axis of 25 ms) to verify the onset detection time point was accurate. The reliability of visually identifying EMG and torque onsets has been previously reported [24]. To determine the reliability of tendon elongation measures, one reviewer retracked tendon elongation for a random sample of 15 trials one week after the initial analysis.

### Statistical analysis and determination of reliability

Data were examined for violations of the assumption of normality (i.e. non-parametric data) using Shapiro-Wilk tests [27]. Non-normal data was log-transformed and re-examined for normality [28]. Data that remained non-parametric following log transformation were examined using non-parametric statistical techniques (involuntary EMD, MVC EMD, normalized MVT, time to tendon force max). Statistical analysis compared the mean dependent variables across levels of ankle joint angles between experimental bouts. Differences between the means for each dependent variable at a particular joint angle were established between testing bouts using paired-samples *t*-tests (parametric variables) and the Wilcoxon Signed-Rank test (non-parametric variables). All statistical analyses were carried out using IBM SPSS (version 26.0, IBM Corporation, Armonk, NY, USA), and statistical significance was set at *P <* 0.05. Test-retest reliability employed both ‘absolute’ and ‘relative’ measures of reliability [27, 29]. The measures examined were percentage difference in the mean (MDiff), intraclass correlation coefficient (ICC; [29]), coefficient of variation (CV; [29]), and Hedges *g* effect size (ES). A two-way random-effects single measures ICC model with absolute agreement was employed. Hedges *g* ES were interpreted as 0 to 0.2 being trivial, 0.2 to 0.6 being small, 0.6 to 1.2 being moderate, and >1.2 as being large [30]. To determine the reliability of variables at each ankle joint angle, reliability indexing was employed using a combination of thresholds, similar to the approach of Joseph et al [31]. Measures were considered to have ‘good’ reliability if ICC scores reached 0.80 or greater, the MDiff score was less than 5%, the CV was 10% or less and the recorded Hedges *g* ES was less than 0.60. ‘Moderate’ reliability was reported as variables that exhibited three of these four criteria. ‘Weak’ reliability was reported as variables that satisfied less than three of these criteria. Additionally, to provide real-world values and enable comparisons with other research, 95% limits of agreement (LoA) [29, 32] were calculated (S1–S8 Tables). An additional analysis was conducted on tendon specific data separated by sex.

## Results

### Angle specific reliability

The reliability of all of the dependent variables separated by ankle angle are displayed in Tables 1–8. The MVT values showed good inter-session reliability across all ankle angles (Table 1). Normalized MVT also demonstrated good reliability at PF ankle angle (Table 1). Despite excellent ICC values (ICC > 0.80), normalized MVT at the AZ and DF angle exhibited only moderate reliability due to MDiff of 5.4% and 6.2% respectively.

**Table 1:** Reliability–maximal plantar flexor strength measures.

		Session		CV (%)	MDiff (%)	Hedges g ES	ICC	Reliability index
		1	2					
MVT (Nm)								
	*PF*	74 ± 18	73 ± 14	4.8	0.4	0.36	0.94	Good
	*AZ*	91 ± 23	87 ± 20	6.0	4.0	0.41	0.91	Good
	*DF*	98 ± 22	94 ± 18	4.7	3.9	0.43	0.91	Good
Norm. MVT (Nm/kg)								
	*PF*	1.12 ± 0.31	1.10 ± 0.23	5.9	1.9	0.02	0.92	Good
	*AZ*	1.38 ± 0.39	1.31 ± 0.30	7.0	5.4	0.25	0.89	Moderate
	*DF*	1.52 ± 0.45	1.43 ± 0.32	8.2	6.2	0.49	0.87	Moderate

Session 1 and Session 2 values reported are mean (±SD). Maximal voluntary torque (MVT); Normalized (Norm.); Coefficient of variation (CV); differences between means (MDiff); effect size (ES); intraclass correlation coefficient (ICC).

**Table 2:** Involuntary measures.

		Session		CV (%)	MDiff (%)	Hedges g ES	ICC	Reliability index
		1	2					
RTDI_0-50_ (Nm.s^-1^)								
	*PF*	41 ± 15	40 ± 12	8.5	2.9	0.20	0.82	Good
	*AZ*	53 ± 8	60 ± 16	9.7	9.1	0.37	0.80	Moderate
	*DF*	94 ± 64	99 ± 55	9.8	4.9	0.17	0.94	Good
RTDI_50-100_ (Nm.s^-1^)								
	*PF*	56 ± 26	60 ± 24	8.6	6.3	0.45	0.94	Moderate
	*AZ*	88 ± 42	88 ± 44	5.4	0.2	0.03	0.98	Moderate
	*DF*	93 ± 23	97 ± 42	10.0	4.5	0.24	0.81	Good
Norm. RTDI_0-50_ (MVT.s^-1^)								
	*PF*	0.63 ± 0.36	0.63 ± 0.26	11.9	0.5	0.12	0.88	Moderate
	*AZ*	0.75 ± 0.26	0.85 ± 0.39	9.2	13.2	0.54	0.87	Moderate
	*DF*	0.84 ± 0.41	0.90 ± 0.40	5.3	7.2	0.57	0.97	Moderate
Norm. RTDI_50-100_ (MVT.s^-1^)								
	*PF*	0.90 ± 0.52	0.92 ± 0.40	20.4	2.7	0.10	0.73	Weak
	*AZ*	0.86 ± 0.30	0.99 ± 0.56	13.2	14.8	0.41	0.71	Weak
	*DF*	0.92 ± 0.34	0.93 ± 0.35	1.6	1.5	0.56	1.00	Good

Session 1 and Session 2 values reported are mean (±SD). Involuntary rate of torque development (RTDI); normalized (Norm.); coefficient of variation (CV), differences between means (MDiff); effect size (ES); intraclass correlation coefficient (ICC)

**Table 3:** Explosive measures.

		Session		CV (%)	MDiff (%)	Hedges g ES	ICC	Reliability index
		1	2					
RTDV_0-50_ (Nm.s^-1^)								
	*PF*	81 ± 31	84 ± 31	8.0	3.0	0.26	0.93	Good
	*AZ*	93 ± 39	96 ± 44	12.1	3.2	0.40	0.87	Moderate
	*DF*	99 ± 37	101 ± 39	15.0	2.4	0.54	0.81	Moderate
RTDV_50-100_ (Nm.s^-1^)								
	*PF*	248 ± 73	286 ± 110	12.4	15.5	0.52	0.73	Weak
	*AZ*	270 ± 104	267 ± 81	13.5	1.1	0.32	0.76	Weak
	*DF*	291 ± 103	286 ± 88	13.3	11.3	0.11	0.85	Weak
RTDV_100-150_ (Nm.s^-1^)								
	*PF*	288 ± 95	301 ± 107	10.4	4.7	0.03	0.89	Moderate
	*AZ*	288 ± 84	314 ± 132	14.0	8.9	0.06	0.78	Weak
	*DF*	334 ± 116	323 ± 121	9.3	3.2	0.07	0.92	Good
Norm. RTDV_0-50_ (MVT.s^-1^)								
	*PF*	1.10 ± 0.41	1.10 ± 0.33	7.3	0.2	0.11	0.93	Good
	*AZ*	1.05 ± 0.33	1.06 ± 0.23	10.4	1.7	0.53	0.75	Weak
	*DF*	0.84 ± 0.31	1.03 ± 0.34	17.5	23.0	0.69	0.80	Weak
Norm. RTDV_50-100_ (MVT.s^-1^)								
	*PF*	3.40 ± 0.74	3.76 ± 0.83	12.1	10.6	0.53	0.73	Weak
	*AZ*	2.89 ± 0.75	3.21 ± 0.86	11.1	11.1	0.21	0.72	Weak
	*DF*	3.03 ± 0.88	3.21 ± 0.78	13.2	5.6	0.06	0.70	Weak
Norm. RTDV_100-150_ (MVT.s^-1^)								
	*PF*	3.89 ± 0.84	3.93 ± 0.89	7.3	0.8	0.15	0.85	Good
	*AZ*	3.12 ± 0.93	3.41 ± 0.97	14.7	9.3	0.07	0.77	Weak
	*DF*	3.14 ± 0.93	3.18 ± 0.96	9.5	1.3	0.23	0.86	Good

Session 1 and Session 2 values reported are mean (±SD). Voluntary rate of torque development (RTDV); normalized (Norm.); coefficient of variation (CV); differences between means (MDiff); effect size (ES); intraclass correlation coefficient (ICC)

**Table 4:** Electromechanical delay.

		Session		CV (%)	MDiff (%)	Hedges g ES	ICC	Reliability index
		1	2					
Involuntary EMD (ms)								
	*PF*	18 ± 4	19 ± 3	5.5	2.1	0.15	0.86	Good
	*AZ*	15 ± 3	15 ± 3	7.0	2.3	0.12	0.85	Good
	*DF*	14 ± 2	13 ± 1	8.5	2.7	0.18	0.82	Good
Explosive EMD (ms)								
	*PF*	31 ± 7	31 ± 10	17.8	1.9	0.06	0.43	Weak
	*AZ*	27 ± 6	23 ± 6	16.9	14.5	0.58	0.37	Weak
	*DF*	28 ± 6	27 ± 7	19.4	5.5	0.38	0.77	Weak
MVC EMD (ms) *								
	*PF*	43 ± 8	43 ± 8	11.0	2.1	0.05	0.47	Weak
	*AZ*	37 ± 9	38 ± 7	14.8	1.5	0.06	0.30	Weak
	*DF*	32 ± 5	31 ± 6	14.0	1.8	0.02	-0.06	Weak
Premotor time (explosive contractions) (ms)								
	*PF*	157 ± 29	173 ± 32	11.4	10.0	0.51	0.46	Weak
	*AZ*	159 ± 21	168 ± 31	11.5	5.9	0.32	0.36	Weak
	*DF*	156 ± 29	158 ± 33	7.8	1.0	0.07	0.42	Moderate

Session 1 and Session 2 values reported are mean (±SD). Electromechanical delay (EMD); Maximal voluntary contraction (MVC); normalized (Norm.); coefficient of variation (CV), differences between means (MDiff); effect size (ES); intraclass correlation coefficient (ICC)

* Log transformed prior to statistical analysis

**Table 5:** Tendon stiffness.

		Session		CV (%)	MDiff (%)	Hedges g ES	ICC	Reliability index
		1	2					
Absolute tendon stiffness (N/mm)								
	*PF*	89 ± 40	85 ± 38	24.8	4.0	0.10	0.60	Weak
	*AZ*	97 ± 35	90 ± 33	20.5	7.5	0.18	0.34	Weak
	*DF*	103 ± 27	99 ± 27	9.6	4.7	0.25	0.76	Moderate

Session 1 and Session 2 values reported are mean (±SD). Coefficient of variation (CV); differences between means (MDiff); effect size (ES); intraclass correlation coefficient (ICC).

**Table 6:** Tendon measures.

		Session		CV (%)	MDiff (%)	Hedges g ES	ICC	Reliability index
		1	2					
Tendon elongation max (mm)								
	*PF*	17 ± 5	17 ± 5	10.2	0.0	0.00	0.83	Moderate
	*AZ*	19 ± 6	19 ± 5	9.4	0.9	0.06	0.85	Good
	*DF*	17 ± 5	18 ± 4	10.6	3.8	0.56	0.88	Moderate
Relative peak strain (%) *								
	*PF*	9.5 ± 3.1	9.4 ± 3.4	13.2	1.2	0.07	0.82	Moderate
	*AZ*	10.3 ± 3.1	10.3 ± 3.1	9.9	0.0	0.09	0.88	Good
	*DF*	9.3 ± 3.1	9.8 ± 2.6	10.3	4.2	0.15	0.83	Moderate
Angle specific peak strain (%)								
	*PF*	9.4 ± 3.0	9.6 ± 3.2	14.6	2.2	0.09	0.77	Weak
	*AZ*	10.4 ± 3.4	10.9 ± 3.9	11.9	5.2	0.27	0.86	Weak
	*DF*	9.8 ± 3.5	10.1 ± 3.0	8.0	2.3	0.26	0.92	Good
Tendon force max (N)								
	*PF*	1210 ± 342	1186 ± 236	6.5	1.9	0.17	0.90	Good
	*AZ*	1504 ± 417	1418 ± 339	6.8	5.7	0.52	0.89	Moderate
	*DF*	1578 ± 405	1524 ± 333	5.0	3.4	0.39	0.93	Good
Norm. tendon force max (N/kg)								
	*PF*	18 ± 6	18 ± 4	6.5	2.0	0.17	0.92	Good
	*AZ*	22 ± 7	21 ± 5	6.8	5.8	0.51	0.90	Moderate
	*DF*	25 ± 9	24 ± 6	6.0	6.1	0.46	0.89	Moderate
Time to tendon force max (s) *								
	*PF*	6.3 ± 1.6	5.7 ± 1.3	15.9	9.6	0.34	0.27	Weak
	*AZ*	5.6 ± 1.0	5.8 ± 1.7	15.9	4.4	0.12	0.26	Weak
	*DF*	6.2 ± 1.0	6.1 ± 0.9	8.7	1.9	0.15	0.65	Moderate
Initial tendon length (cm)								
	*PF*	18.2 ± 2.0	18.7 ± 2.7	4.7	2.7	0.36	0.84	Good
	*AZ*	18.4 ± 2.0	19.1 ± 3.1	5.6	3.8	0.43	0.81	Good
	*DF*	18.8 ± 1.9	19.5 ± 3.0	5.4	3.6	0.39	0.78	Moderate

Session 1 and Session 2 values reported are mean (±SD). Normalized (Norm.); coefficient of variation (CV); differences between means (MDiff); effect size (ES); intraclass correlation coefficient (ICC)

* Log transformed prior to statistical analysis

**Table 7:** Female tendon and maximal strength measures.

		Session		CV (%)	MDiff (%)	Hedges g ES	ICC	Reliability index
		1	2					
MVT (Nm)								
	*PF*	76 ± 15	75 ± 12	4.6	1.3	0.17	0.96	Good
	*AZ*	94 ± 20	90 ± 21	3.8	4.1	0.42	0.95	Good
	*DF*	92 ± 5	91 ± 6	3.2	1.9	0.17	0.91	Good
Normalized MVT (Nm/kg)								
	*PF*	1.25 ± 0.32	1.20 ± 0.19	14.1	4.2	0.32	0.93	Moderate
	*AZ*	1.53 ± 0.40	1.43 ± 0.33	7.8	6.8	0.59	0.93	Moderate
	*DF*	1.62 ± 0.55	1.51 ± 0.38	7.4	6.2	0.42	0.92	Moderate
Absolute stiffness (N/mm)								
	*PF*	103 ± 50	102 ± 34	16.2	1.0	0.03	0.71	Weak
	*AZ*	103 ± 22	100 ± 30	12.9	2.8	0.08	0.20	Weak
	*DF*	100 ± 15	99 ± 19	5.2	4.3	0.32	0.80	Good
Tendon elongation max (mm)								
	*PF*	16 ± 5	15 ± 5	5.5	1.9	0.08	0.86	Good
	*AZ*	17 ± 6	18 ± 5	8.4	2.9	0.23	0.96	Good
	*DF*	16 ± 6	18 ± 4	9.3	9.5	0.58	0.85	Moderate
Relative peak strain (%) *								
	*PF*	9.5 ± 3.4	9.3 ± 4.0	7.5	2.6	0.10	0.90	Good
	*AZ*	10.3 ± 3.6	10.9 ± 4.5	5.3	5.9	0.38	0.96	Moderate
	*DF*	9.9 ± 4.2	10.8 ± 3.5	9.3	12.0	0.52	0.92	Moderate
Angle specific peak strain (%)								
	*PF*	9.0 ± 3.2	9.6 ± 3.6	10.8	5.9	0.14	0.72	Weak
	*AZ*	10.1 ± 4.2	11.2 ± 4.7	11.4	10.4	0.58	0.85	Weak
	*DF*	10.3 ± 4.9	10.6 ± 4.2	9.8	6.7	0.47	0.97	Moderate
Tendon force max (N)								
	*PF*	1318 ± 309	1264 ± 208	6.8	4.1	0.42	0.94	Good
	*AZ*	1594 ± 352	1489 ± 329	3.7	6.5	0.56	0.92	Moderate
	*DF*	1520 ± 111	1489 ± 192	3.4	2.1	0.18	0.92	Good
Norm. tendon force max (N/kg)								
	*PF*	21 ± 6	20 ± 4	8.4	4.4	0.41	0.95	Good
	*AZ*	25 ± 7	23 ± 6	6.2	6.8	0.59	0.93	Moderate
	*DF*	27 ± 10	25 ± 7	3.6	7.2	0.43	0.93	Moderate
Initial tendon length (cm)								
	*PF*	19.2 ± 1.5	20.9 ± 1.9	5.2	7.7	0.61	0.81	Weak
	*AZ*	19.1 ± 1.3	21.0 ± 2.1	6.2	8.4	0.61	0.79	Weak
	*DF*	19.2 ± 2.0	21.4 ± 2.3	5.6	10.2	0.62	0.76	Weak

Session 1 and Session 2 values reported are mean (±SD). Maximal voluntary torque (MVT); Normalized (Norm.); coefficient of variation (CV); differences between means (MDiff); effect size (ES); intraclass correlation coefficient (ICC)

* Log transformed prior to statistical analysis

**Table 8:** Male tendon and maximal strength measures.

		Session		CV (%)	MDiff (%)	Hedges g ES	ICC	Reliability index
		1	2					
MVT (Nm)								
	*PF*	71 ± 16	73 ± 14	4.0	0.6	0.06	0.97	Good
	*AZ*	88 ± 23	87 ± 20	6.0	3.9	0.36	0.96	Good
	*DF*	103 ± 20	94 ± 16	6.3	4.3	0.78	0.98	Moderate
Normalized MVT (Nm)								
	*PF*	0.98 ± 0.22	1.00 ± 0.23	5.1	1.4	0.15	0.97	Good
	*AZ*	1.21 ± 0.31	1.17 ± 0.22	7.1	3.2	0.32	0.95	Good
	*DF*	1.42 ± 0.33	1.35 ± 0.26	8.0	4.2	0.69	0.97	Moderate
Absolute stiffness (N/mm)								
	*PF*	72 ± 25	66 ± 38	26.1	8.9	0.19	0.64	Weak
	*AZ*	91 ± 50	78 ± 37	18.4	20.5	0.26	0.58	Weak
	*DF*	106 ± 41	98 ± 34	9.0	9.6	0.56	0.97	Moderate
Tendon elongation max (mm)								
	*PF*	18 ± 6	18 ± 5	12.2	1.2	0.07	0.93	Moderate
	*AZ*	20 ± 6	20 ± 5	8.5	1.0	0.05	0.86	Good
	*DF*	18 ± 5	18 ± 3	14.9	0.3	0.04	0.96	Moderate
Relative peak strain (%) *								
	*PF*	9.5 ± 3.1	9.5 ± 3.0	16.7	0.2	0.01	0.93	Moderate
	*AZ*	10.4 ± 2.7	10.4 ± 3.3	9.8	0.2	0.00	0.90	Good
	*DF*	9.2 ± 2.2	8.9 ± 1.2	12.9	2.5	0.18	0.87	Moderate
Angle specific peak strain (%)								
	*PF*	9.5 ± 3.1	9.5 ± 3.0	18.1	0.2	0.01	0.93	Moderate
	*AZ*	10.7 ± 2.8	10.7 ± 3.2	13.0	0.2	0.01	0.88	Moderate
	*DF*	9.5 ± 2.2	9.3 ± 1.3	8.8	1.7	0.13	0.87	Good
Tendon force max (N)								
	*PF*	1058 ± 359	1077 ± 250	5.1	1.8	0.13	0.95	Good
	*AZ*	1378 ± 508	1318 ± 363	7.1	4.3	0.37	0.97	Good
	*DF*	1636 ± 591	1559 ± 488	6.4	4.7	0.68	0.99	Moderate
Norm.tendon force max (N/kg)								
	*PF*	15 ± 4	15 ± 3	5.1	2.7	0.20	0.94	Good
	*AZ*	19 ± 6	18 ± 4	7.1	3.8	0.34	0.96	Good
	*DF*	23 ± 6	22 ± 5	8.0	4.5	0.69	0.98	Moderate
Initial tendon length (cm)								
	*PF*	17.2 ± 1.7	17.2 ± 2.7	4.5	0.0	0.01	0.89	Good
	*AZ*	17.6 ± 1.7	17.1 ± 3.2	5.8	0.3	0.03	0.89	Good
	*DF*	18.1 ± 1.7	18.0 ± 2.9	5.5	0.4	0.04	0.80	Good

Session 1 and Session 2 values reported are mean (±SD). Maximal voluntary torque (MVT); Normalized (Norm.); coefficient of variation (CV); differences between means (MDiff); effect size (ES); intraclass correlation coefficient (ICC)

* Log transformed prior to statistical analysis

Across ankle angles, RTDI_0-50_ and RTDI_50-100_ produced acceptable ICC’s (ICC > 0.80), small CV (5.4–10.0%), and small Hedges *g* ES (0.03–0.45). RTDI at the DF angle was the most reliable of the ankle angles, with good reliability for RTDI_0-50_, and RTDI_50-100_ time periods (Table 2). Normalized RTDI_0-50_ had moderate reliability at all ankle angles (Table 2). Similar to RTDI, normalized RTDI at the DF ankle angle was the most reliable of the ankle angles with moderate reliability for RTDI_0-50_ and good reliability RTDI_50-100_. Normalized RTDI_50-100_ had weak reliability for both PF and AZ angles (Table 2). For electrically evoked twitch contractions, one participant did not complete the evoked twitch contractions, thus results are reported as *n* = 13. Across ankle angles, RTDV_0-50_ showed good reliability at PF ankle angle and moderate reliability at AZ and DF ankle angles (Table 3). RTDV_50-100_ was less consistent, with weak reliability exhibited for all ankle angles (Table 3). Reliability of RTDV_100-150_ varied across ankle angles, with good reliability at AZ ankle angle, moderate reliability at PF ankle angle, and weak reliability at DF ankle angle (Table 3). Normalized RTDV_0-50_ at the PF ankle angle exhibited good reliability (Table 3). Also, normalized RTDV_100-150_ at the PF and DF ankle angle showed good reliability (Table 3). The remaining normalized RTDV at all time periods (0–50, 50–100, 100–150 ms) and ankle angles demonstrated weak reliability. In general, the explosive measures of RTDV (Table 3) were less reliable compared to the electrically evoked RTDI measures (Table 2).

Involuntary EMD demonstrated good inter-day reliability across the three ankle angles (Table 4). Explosive and maximal voluntary contraction EMD were less reliable, with weak reliability across ankle angles (Table 4).

For tendon elongation tracking, good intra-rater reliability (TE = 0.44 mm; ICC = 0.98) was observed. Absolute tendon stiffness at the DF joint angle exhibited the best inter-session reliability (Table 5) with the narrowest LoA compared to other joint angles (absolute = -27 to +32 N/mm). Inter-session reliability of tendon measures used in the calculation of absolute stiffness are displayed in Table 6.

### Sex-specific reliability of tendon measures

Males and females demonstrated similar inter-session reliability for absolute tendon stiffness with the DF joint angle exhibiting the best reliability for both (Tables 7, 8). Initial tendon length measured at each ankle angle prior to the maximal voluntary contractions demonstrated contrasting reliability across sexes (Tables 7, 8). Males had good reliability across joint angles with no significant differences between the session means across joint angles (Table 8). However, females demonstrated weak reliability across joint angles (ICC = 0.76–0.81, CV = 5.2–6.2%, MDiff = 7.7–10.2%, ES = 0.61–0.62), and significant differences between the session means at each level of joint angle (*P* = 0.004–0.023).

## Discussion

This study assessed the inter-session reliability of plantar flexor mechanical properties across ankle angles and sex. Our results demonstrated that: i) MVT and normalized MVT is reliable across ankle angles; ii) RTDI and normalized RTDI measures were reliable across time periods and ankle angles, except for normalized RTDI_50-100_ at the PF and AZ ankle angle which was highly variable; and iii) RTDV_0-50_ was reliable on a group level between testing sessions, however, showed greater within-participant variability from 50 ms onward; iv) involuntary EMD is the most reliable EMD measure across ankle angles; v) the reliability of tendon stiffness is best at the DF ankle angle Considering sex-based differences in the reliability of tendon measures found that females had significantly different initial tendon length across ankle angles between testing sessions. Despite this, tendon excursion, force, and stiffness measures demonstrated similar reliability compared to males.

Assessment of muscle strength through MVT demonstrated good reliability (ICC > 0.9, CV < 6%), and normalized MVT also demonstrated moderate-to-good reliability (ICC = > 0.8, CV = < 10%). MVT values across ankle angles and sessions (PF– 74/73 Nm, AZ– 91/87 Nm, DF– 98/94 Nm) aligned with MVT values from previous research assessing similar populations [22, 33]. Similar findings on plantar flexor MVT reliability have been reported by Merlet et al [10] (ICC = 0.77–0.88; with varying knee angles), and Chen et al. [13] (ICC = 0.96–0.98; ankle angles of 20° PF, 0°, 20° DF) with both these studies separating test sessions by one week. Further research has demonstrated high reliability of MVT from Stutzig et al. [2] with two weeks separating test sessions (ICC = 0.92; ankle angle of 90) and Clark et al. [1] with four weeks apart (ICC = 0.97), which suggests high reliability of these measurements over longer test-retest time periods. The findings on the reliability of MVT across ankle angles has important implications for the assessment of MVT in research and practical settings. According to the results, MVT can be confidently assessed and compared between testing sessions across ankle angles measured in the present study.

Evaluation of RTD through electrically evoked involuntary, and explosive voluntary contractions reveal the relative contribution of neural and muscular factors to performance during rapid contractions [12]. Previous research highlighted that RTDV is influenced by joint angle due to changes in both neuromuscular activation and the contractile response [34]. This study aimed to establish the reliability of RTDI and RTDV across joint angles. The reliability of RTDI_0-50_ and RTDV_0-50_ was highly reproducible on a group level (ICC > 0.80) with moderate within-participant reliability (CV = 8.0–15.0%) across ankle angles. RTDI_50-100_ exhibited moderate-to-good reliability (ICC > 0.80, CV < 10%), however RTDV was less reliable across ankle angles (Table 3). RTDV_100-150_ demonstrated consistent group values across the sessions (ICC = 0.78–0.92), but with variable levels of within-participant reliability across ankle angles (CV = 9.3–14.0%). The results demonstrate better reliability for early phase RTDI and RTDV across ankle angles. Similar levels of reliability for plantar flexor early-phase for RTDI and RTDV has been previously reported [1]. Across ankle angles and contraction types, RTD_0-50_ exhibited the best reliability. The normalization of RTD to angle-specific MVT tended to reduce the reliability across ankle angles. While some iterations of normalized RTDI and RTDV exhibited moderate-to-good reliability, the tendency was for normalized RTD measures to demonstrate weak reliability as seen for normalized RTDI (Table 2) and normalized RTDV (Table 3). The reason for the varied reliability across these normalized measures is unclear. Despite this, the normalization of torque-time curve measures to a reference specific torque (i.e. MVT) at the specific testing angle remains important [25]. Evoked and voluntary torque-time curves are determined by contraction-specific joint kinematics, thus normalization is recommended if comparing results between contraction types and joint angles [25].

For EMD, involuntary was the most stable measure with good reliability across ankle angles (ICC > 0.8, CV <10%). The results are in line with previously reported reliability values of plantar flexor involuntary EMD [35]. The EMD values from voluntary, explosive, and maximal contractions were less stable, exhibiting weak reliability across ankle angles (Table 4). The different muscle activation strategies between voluntary and involuntary EMD could explain the tendency for voluntary EMD measures to be less reliable. Voluntary EMD measures include a large neural component, which can introduce significant variability to the EMG and force onset determination, a key part of EMD. Additionally, there are several factors that influence EMG signals during voluntary contractions which may affect the reliability of voluntary EMD measures through detection errors. Type I error (identifying a muscle as excited when it is not) due to crosstalk is common when assessing thin superficial muscles as the deeper muscle’s EMG activity could outweigh that of the superficial muscle leading to detection errors. Type II error (identifying a muscle as not excited when it is) is also a concern and can result from a single bipolar EMG sampling a large muscle. Superficial fiber activation of a muscle is the main contributor to the EMG signal, thus activation of deeper fibers of that muscle may not be sampled by the EMG sensor, leading to missed onset detections [36].

Limited research has examined the influence of different ankle angles on tendon stiffness and in particular, the reliability of the measure. The current study suggests measuring tendon stiffness at the DF angle improves reliability on a group and individual level. Further examination of the components that contribute to the calculation of tendon stiffness (Table 6) show that time to maximum tendon force had improved inter-day reliability at the DF (ICC = 0.65, CV = 8.7%) ankle angle compared to other angles (PF: ICC = 0.27, CV = 15.9%; AZ: ICC = 0.26, CV = 15.9%). The time to maximum tendon force is a factor in the calculation of tendon strain rate, and tendon strain rate is known to influence calculated tendon stiffness [37]. Thus, improved inter-session reliability of this component of tendon strain rate may contribute to the improved reliability of tendon stiffness measures at the DF ankle angle. Despite tendon stiffness demonstrating moderate-to-good reliability at the DF angle, the LoA for absolute stiffness (27 to +32 N/mm) are wide. A potential explanation for this variability is that tendon stiffness calculation can be influenced by several factors including measurement equipment, inherent individual variation, and methodological issues e.g. tendon moment arm measurement and ankle joint rotation [38]. For future studies, examining tendon stiffness at the DF ankle angle is recommended. Tendon stiffness at this ankle angle demonstrates improved inter-session reliability, potentially due to a participant’s ability to produce a more uniform tendon strain rate. This could result from the removal of the toe region of the force/elongation and force/strain curve resulting in immediate tendon elongation at this joint angle.

A secondary study aim was to examine any sex-related differences in the reliability of tendon measures. Greater compliance in the connective tissue of females compared to males has been attributed to differences in hormonal composition between the sexes [39]. Controlling for these hormonal differences using the oral contraceptive pill, to regulate estrogen and progesterone levels, similar reliability in tendon measures across the sexes was expected. While tendon excursion, force, and stiffness measures demonstrated similar reliability across sex, initial tendon length was greater across ankle angles for females on the second day of testing and demonstrated weak reliability (Table 7). The similar levels of tendon excursion during an active contraction in oral contraceptive pill users is similar to previous research [39], however, the greater compliance in the female tendon at rest is novel. While oral contraceptive use ensures stability in the circulating levels of estrogen and progesterone, differences in the type and brand of pill could be of importance as the concentration of these hormones varies across types and brands of pill [40]. However, in the absence of blood measures to examine the impact of circulating hormones on tendon laxity, this hypothesis cannot be confirmed. An additional consideration which may explain the differences in female resting tendon length is the standardisation of the measurement of this mechanical property. Resting tendon length was quantified as the length of the linear distance between the insertion of the tendon on the calcaneal tuberosity and the MG muscle-tendon junction with the knee at 180° and the ankle at 20° PF, as at this joint angle the passive moment is almost zero [23]. While the participants were instructed to relax their leg muscles, differences in low-level force being applied to the tendon across participants and between testing sessions may have impacted the standardization of measuring resting tendon length. This measurement issue potentially provides a more reasonable explanation for the differences observed for females rather than a true biological difference between testing sessions, as mechanical properties should remain the same between sessions, particularly when these hormonal differences were controlled for by investigating females using the oral contraceptive pill. Potentially, future assessments of resting tendon length would benefit from adopting a standardized approach of measuring resting tendon length based on participants applying a low level of force to the tendon (e.g. 50/100 N). Similar to the amalgamated group analysis, the DF angle produced the best reliability when assessing tendon properties across the respective sexes. Further investigation to confirm these results is encouraged due to the small sample size when divided into groups based on sex (*n* = 7). The initial analysis suggests oral contraceptive use can regulate the influence of fluctuating hormones on tendon tissue compliance and results can thus be reliably interpreted when comparing sexes.

### Practical applications

Assessing the reliability of plantar flexor mechanical measures across joint angles allows researchers to identify the measurement angles that can be reliably interpreted to measure a ‘real change’ due to intervention or for between-group comparisons. With moderate to good reliability demonstrated, the MVT, involuntary RTD, involuntary EMD, tendon force, tendon elongation, and tendon relative peak strain represented the most reliable parameters. The findings demonstrate that these mechanical parameters of the plantar flexor muscle-tendon unit can be assessed and compared across testing sessions irrespective of the ankle angle employed. Many previous studies examined reliability of mechanical measures on consecutive days, however in the current study we examined this over time. Practically, this could be of relevance for intervention studies conducted over several weeks. However, future studies should investigate the reliability of longer time periods between sessions, as many intervention studies are long-term (i.e. > 10 days).

### Limitations paragraph

There are limitations of note in this study that need to be addressed. Firstly, caution should be approached in inferring the findings of the current study on the plantar flexors to other muscle groups as this could vary between muscles. Additionally, caution is advised in extrapolating these findings to other populations. The participants in this study were young, physically active individuals. As a result, applying these to other populations where physical characteristics could influence the examined mechanical variables is not advised. The contractions in the study were performed across three ankle joint angles with the knee angle kept consistent (180°). Muscle length is known to influence force output [41] and M-wave [42]. While the participants ankle and knee were secured, small changes in joint angle of either the ankle or knee may induce changes in muscle length which could impact the mechanical parameters measured. Within sessions, any trials that exceeded >2° from the ankle testing angle during measurement was repeated, and knee angle was monitored throughout trials. Between sessions the participants position on the dynamometer was documented to replicate this position across the sessions. Despite this, it cannot be ruled out that changes in muscle length of participants within and between sessions may have impacted the measured mechanical parameters. Finally, the sample size in the current study is a limitation. While the sample size is similar to sample sizes of previous research examining the influence of joint position on the reliability of mechanical variables [2, 13], it is not sufficient to provide definitive conclusions. The findings of this study should be considered as a pilot study with further research required with a larger sample.

## Conclusion

Prior to this study, the effect of ankle angle on the reliability of mechanical measures of the plantar flexor muscle-tendon unit was undetermined. In conclusion, MVT measures were reliable across ankle angles. RTD measures (involuntary, and voluntary explosive) showed good group level reliability and moderate reliability for an individual during the early phase of contraction across ankle angles. From 50 ms onward, involuntary evoked torque production demonstrated moderate-to-good reliability. Explosive voluntary torque measures tended to be less reliable from 50 ms onward, and varied reliability across angles for late-phase torque development. Tendon stiffness demonstrated the best reliability at the DF angle. Sex-based differences in the reliability of tendon measures found that females had significantly greater initial tendon length between testing sessions, potentially related to the impact of circulating hormones on these tissues, or more likely, the result of a measurement issue. Despite this, tendon excursion, force, and stiffness measures demonstrated similar reliability compared to males. The findings of this study highlight that ankle angle changes can influence the reliability of mechanical measures of the plantar flexors, which should be considered for future studies to attain reliable outcomes.

## Supporting information

S1 Table EMD measures and LoA. (PDF)

S2 Table Tendon Stiffness measures and LoA. (PDF)

S3 Table Tendon measures and LoA. (PDF)

S4 Table Maximal voluntary contraction and muscle thickness measures and LoA. (PDF)

S5 Table Involuntary RTD measures and LoA. (PDF)

S6 Table Involuntary absolute torque measures and LoA. (PDF)

S7 Table Explosive voluntary RTD measures and LoA. (PDF)

S8 Table Explosive voluntary RTD measures and LoA. (PDF)
