# Supplementary material for: Reliability of mechanical properties of the plantar flexor muscle tendon unit with consideration to joint angle and sex
Source: PLoS One. 2023 Jun 23;18(6):e0287431. doi: 10.1371/journal.pone.0287431 (PMC10289375; doi:10.1371/journal.pone.0287431)
Supplement: S1 Table — (PDF) [file pone.0287431.s001.pdf]

S1 Table. EMD measures and LoA

	Mean (±s)				Limits of agreement (ms)	
	Day 1		Day 2		LloA	UloA
**Involuntary (ms)**						
PF	18.25	3.68	18.63	2.94	-3.33	3.87
AZ	14.46	3.28	14.79	2.50	-2.99	3.47
DF	13.51	2.29	13.15	1.36	-3.52	4.25
**Explosive (ms)**						
PF	31.38	7.00	30.80	10.20	-18.05	19.19
AZ	26.90	6.00	23.00	5.86	-8.85	16.20
DF	28.20	5.58	26.64	7.20	-6.74	10.02
**Maximal voluntary contraction (ms)**						
PF	42.48	8.44	43.36	7.59	-15.51	17.27
AZ	37.24	9.00	37.80	7.39	-18.85	20.00
DF	31.96	4.80	31.38	6.13	-15.59	15.93
**Premotor time (ms)**						
PF	157.36	28.84	172.96	32.37	-44.15	75.37
AZ	159.00	21.10	168.39	30.56	-48.49	67.27
DF	156.00	29.00	157.48	33.05	-40.22	43.18
