# Supplementary material for: Reliability of mechanical properties of the plantar flexor muscle tendon unit with consideration to joint angle and sex
Source: PLoS One. 2023 Jun 23;18(6):e0287431. doi: 10.1371/journal.pone.0287431 (PMC10289375; doi:10.1371/journal.pone.0287431)
Supplement: S2 Table — (PDF) [file pone.0287431.s002.pdf]

S2 Table. Tendon Stiffness measures and LoA

	Mean (±s)				Limits of agreement	
	**Day 1**		**Day 2**		**LloA**	**UloA**
**Absolute stiffness (N/mm)**						
PF	88.85	40.1	85.34	37.6	-69.04	66.07
AZ	96.99	34.7	89.68	32.7	-72.23	76.85
DF	103.30	27.2	98.8	26.7	-26.80	32.13
