# Supplementary material for: Reliability of mechanical properties of the plantar flexor muscle tendon unit with consideration to joint angle and sex
Source: PLoS One. 2023 Jun 23;18(6):e0287431. doi: 10.1371/journal.pone.0287431 (PMC10289375; doi:10.1371/journal.pone.0287431)
Supplement: S3 Table — (PDF) [file pone.0287431.s003.pdf]

S3 Table. Tendon measures and LoA

		Mean (±s)				Limits of agreement	
		Day 1		Day 2		LloA	UloA
**Elongation max (mm)**							
	PF	16.92	5.33	16.91	5.02	-6.14	6.15
	AZ	18.58	5.68	18.74	4.66	-5.67	6.00
	DF	16.98	5.00	18.47	3.65	-3.73	6.71
**Relative peak strain (%)**							
	PF	9.50	3.10	9.38	3.36	-3.77	4.00
	AZ	10.33	3.05	10.33	3.05	-3.10	3.72
	DF	9.32	3.10	9.80	2.59	-2.80	3.76
**Absolute peak strain (%)**							
	PF	9.35	3.01	9.55	3.17	-4.01	4.41
	AZ	10.40	3.38	10.94	3.86	-3.30	4.37
	DF	9.83	3.49	10.06	2.96	-2.47	2.92
**Tendon force max. (N)**							
	PF	1209.60	342.18	1186.35	235.54	-238.11	284.60
	AZ	1503.55	416.66	1418.05	338.54	-234.60	405.60
	DF	1577.86	405.25	1523.74	333.23	-217.92	326.16
**Normalised tendon force max**							
	PF	18.09	6.03	17.73	4.23	-3.82	4.55
	AZ	22.44	6.81	21.15	5.42	-3.67	6.26
	DF	24.98	8.63	23.47	6.12	-5.06	8.10
**Time to tendon force max (s)**							
	PF	6.25	1.60	5.65	1.33	-2.88	4.07
	AZ	5.55	1.04	5.80	1.66	-3.10	3.58
	DF	6.23	0.97	6.11	0.87	-1.61	1.85
**Resting tendon length (cm)**							
	PF	18.18	1.95	18.67	2.65	-2.21	3.20
	AZ	18.38	1.97	19.08	3.06	-2.47	3.86
	DF	18.83	1.93	19.50	2.96	-2.72	4.07
