# Supplementary material for: Reliability of mechanical properties of the plantar flexor muscle tendon unit with consideration to joint angle and sex
Source: PLoS One. 2023 Jun 23;18(6):e0287431. doi: 10.1371/journal.pone.0287431 (PMC10289375; doi:10.1371/journal.pone.0287431)
Supplement: S4 Table — (PDF) [file pone.0287431.s004.pdf]

S4 Table. Maximal voluntary contraction and muscle thickness measures and LoA

	Mean (±s)		Limits of agreement	
	**Day 1**	**Day 2**	**LloA**	**UloA**
**Maximal voluntary contraction (Nm)**				
PF	74	73.41	-11.30	8.86
	17.85	14.27		
AZ	90.64	87.03	-13.74	15.96
	23.13	19.62		
DF	97.84	94.07	-12.85	15.39
	22.23	18.24		
**Normalised maximal voluntary torque (Nm/kg)**				
PF	1.12	1.10	-0.20	0.21
	0.31	0.23		
AZ	1.38	1.31	-0.22	0.31
	0.39	0.30		
DF	1.52	1.43	-0.36	0.28
	0.45	0.32		
**Muscle thickness (mm)**	21.28	20.93	-0.30	0.88
