# Supplementary material for: Reliability of mechanical properties of the plantar flexor muscle tendon unit with consideration to joint angle and sex
Source: PLoS One. 2023 Jun 23;18(6):e0287431. doi: 10.1371/journal.pone.0287431 (PMC10289375; doi:10.1371/journal.pone.0287431)
Supplement: S5 Table — (PDF) [file pone.0287431.s005.pdf]

S5 Table. Involuntary RTD measures and LoA

		Mean (±s)				Limits of agreement	
		Day 1		Day 2		LloA	UloA
**RTD 0-50 ms (N.s-1)**							
	PF	41.160	14.917	39.953	12.386	-15.964	18.376
	AZ	52.700	8.032	59.595	15.988	-14.905	28.697
	DF	93.622	64.283	98.922	54.962	-33.783	44.383
**RTD 50-100 ms**							
	PF	56.089	25.895	59.602	23.501	-11.915	18.940
	AZ	88.084	41.641	87.877	43.631	-13.844	14.258
	DF	92.723	23.417	96.906	41.835	-29.459	37.824
**Norm. RTD 0-50 ms**							
	PF	0.629	0.356	0.632	0.255	-0.297	0.302
	AZ	0.754	0.258	0.854	0.385	-0.208	0.407
	DF	0.837	0.405	0.897	0.399	-0.101	0.221
**Norm. RTD 50-100 ms**							
	PF	0.900	0.524	0.924	0.404	-0.666	0.714
	AZ	0.863	0.298	0.990	0.556	-0.481	0.735
	DF	0.919	0.343	0.932	0.352	-0.034	0.062
