# Supplementary material for: Reliability of mechanical properties of the plantar flexor muscle tendon unit with consideration to joint angle and sex
Source: PLoS One. 2023 Jun 23;18(6):e0287431. doi: 10.1371/journal.pone.0287431 (PMC10289375; doi:10.1371/journal.pone.0287431)
Supplement: S6 Table — (PDF) [file pone.0287431.s006.pdf]

S6 Table. Involuntary absolute torque measures and LoA

		Mean (±s)				Limits of agreement	
		Day 1		Day 2		LloA	UloA
**Maximum torque**							
	PF	6.29	3.11	6.97	2.98	-4.67	6.04
	AZ	11.00	5.62	11.72	4.84	-6.55	8.00
	DF	15.79	8.25	17.25	6.09	-10.18	13.10
**Time to maximum torque**							
	PF	0.13	0.01	0.13	0.02	-0.02	0.02
	AZ	0.13	0.02	0.14	0.02	-0.02	0.02
	DF	0.14	0.02	0.15	0.02	-0.02	0.02
**Absolute torque 25 ms**							
	PF	0.47	0.12	0.47	0.13	-0.18	0.19
	AZ	0.73	0.23	0.74	0.22	-0.31	0.32
	DF	0.94	0.28	1.10	0.62	-0.40	0.71
**Absolute torque 50 ms**							
	PF	2.07	0.66	1.88	0.62	-0.46	0.83
	AZ	4.17	1.89	4.50	1.69	-0.71	1.38
	DF	5.88	3.34	5.42	2.36	-0.59	1.53
**Absolute torque 75 ms**							
	PF	4.59	2.67	4.18	1.94	-1.56	2.38
	AZ	7.04	3.36	7.35	3.33	-2.50	3.10
	DF	9.07	4.39	9.66	4.46	-4.54	5.70
**Absolute torque 100 ms**							
	PF	5.15	2.11	5.10	1.52	-1.93	2.05
	AZ	9.84	5.75	9.15	3.59	-4.74	6.12
	DF	11.93	5.70	12.80	5.85	-5.28	7.02

		Mean (±s)				Limits of agreement	
		Day 1		Day 2		LloA	UloA
**Norm. absolute torque 25 ms**							
	PF	0.007	0.004	0.007	0.003	-0.004	0.005
	AZ	0.010	0.004	0.011	0.004	-0.003	0.003
	DF	0.010	0.005	0.012	0.006	-0.004	0.008
**Norm. absolute torque 50 ms**							
	PF	0.026	0.012	0.026	0.011	-0.013	0.013
	AZ	0.042	0.016	0.044	0.018	-0.017	0.021
	DF	0.043	0.018	0.051	0.024	-0.014	0.031
**Norm. absolute torque 75 ms**							
	PF	0.047	0.020	0.046	0.020	-0.018	0.019
	AZ	0.076	0.031	0.082	0.034	-0.033	0.045
	DF	0.084	0.037	0.091	0.039	-0.051	0.066
**Norm. absolute torque 100 ms**							
	PF	0.058	0.030	0.060	0.031	-0.013	0.017
	AZ	0.093	0.050	0.099	0.047	-0.045	0.057
	DF	0.096	0.035	0.119	0.057	-0.026	0.072
