# Supplementary material for: Reliability of mechanical properties of the plantar flexor muscle tendon unit with consideration to joint angle and sex
Source: PLoS One. 2023 Jun 23;18(6):e0287431. doi: 10.1371/journal.pone.0287431 (PMC10289375; doi:10.1371/journal.pone.0287431)
Supplement: S7 Table — (PDF) [file pone.0287431.s007.pdf]

S7 Table. Explosive voluntary RTD measures and LoA

		Mean (±s)				Limits of agreement	
		Day 1		Day 2		LloA	UloA
**RTD 0-50 ms**							
	PF	81.27	30.67	83.73	31.12	-21.21	27.76
	AZ	92.84	39.19	95.85	43.66	-34.03	50.40
	DF	98.90	36.46	101.22	39.35	-31.89	55.84
**RTD 50-100 ms**							
	PF	247.46	72.45	285.92	109.98	-57.51	116.47
	AZ	269.58	103.65	266.57	80.46	-93.50	130.20
	DF	291.31	102.62	285.98	87.92	-95.24	106.10
**RTD 100-150 ms**							
	PF	287.53	94.91	301.16	107.30	-99.22	92.22
	AZ	288.38	84.42	313.90	132.26	-137.48	135.40
	DF	333.96	115.96	323.29	121.02	-96.71	93.41
**Norm. RTD 0-50 ms**							
	PF	1.10	0.41	1.10	0.33	-0.27	0.31
	AZ	1.05	0.33	1.06	0.23	-0.23	0.40
	DF	0.84	0.31	1.03	0.34	-0.23	0.47
**Norm. RTD 50-100 ms**							
	PF	3.40	0.74	3.76	0.83	-0.85	1.47
	AZ	2.89	0.75	3.21	0.86	-1.06	1.32
	DF	3.03	0.88	3.21	0.78	-1.17	1.25
**Norm. RTD 100-150 ms**							
	PF	3.89	0.84	3.93	0.89	-0.97	1.13
	AZ	3.12	0.93	3.41	0.97	-1.34	1.43
	DF	3.14	0.93	3.18	0.96	-0.96	1.21
