# Supplementary material for: Reliability of mechanical properties of the plantar flexor muscle tendon unit with consideration to joint angle and sex
Source: PLoS One. 2023 Jun 23;18(6):e0287431. doi: 10.1371/journal.pone.0287431 (PMC10289375; doi:10.1371/journal.pone.0287431)
Supplement: S8 Table — (PDF) [file pone.0287431.s008.pdf]

S8 Table. Explosive voluntary RTD measures and LoA

		Mean (±s)				Limits of agreement	
		Day 1		Day 2		LloA	UloA
**Absolute torque 25 ms**							
	PF	0.903	0.410	0.956	0.304	-0.393	0.498
	AZ	1.232	0.587	1.190	0.576	-0.779	0.866
	DF	1.912	1.463	1.733	0.973	-1.669	1.725
**Absolute torque 50 ms**							
	PF	3.866	1.588	4.213	1.483	-1.466	2.160
	AZ	5.121	2.170	5.167	2.331	-2.309	2.697
	DF	6.297	2.518	6.452	3.157	-3.106	4.651
**Absolute torque 75 ms**							
	PF	9.352	3.459	10.471	3.805	-3.615	5.852
	AZ	11.515	4.648	12.016	5.498	-4.439	5.790
	DF	12.659	4.286	15.643	8.865	-8.039	14.697
**Absolute torque 100 ms**							
	PF	16.475	5.677	18.597	6.20	-6.254	10.498
	AZ	18.451	20.971	20.637	9.214	-7.533	11.905
	DF	20.971	6.604	21.553	7.757	-9.004	13.040
**Absolute torque 125 ms**							
	PF	24.518	7.583	26.784	9.333	-8.453	13.186
	AZ	25.197	8.323	29.342	15.572	-8.541	11.047
	DF	30.503	8.565	30.120	10.643	-11.498	15.097
**Absolute torque 150 ms**							
	PF	30.102	9.899	33.698	11.579	-9.795	16.987
	AZ	32.803	12.138	33.788	10.751	-10.993	16.622
	DF	36.896	11.633	37.664	13.563	-13.246	19.895

		Mean (±s)				Limits of agreement	
		Day 1		Day 2		LloA	UloA
**Norm. absolute torque 25 ms**							
	PF	0.013	0.053	0.013	0.004	-0.005	0.005
	AZ	0.011	0.004	0.013	0.021	-0.008	0.011
	DF	0.016	0.012	0.021	0.011	-0.014	0.026
**Norm. absolute torque 50 ms**							
	PF	0.054	0.022	0.055	0.017	-0.017	0.020
	AZ	0.048	0.014	0.053	0.012	-0.024	0.036
	DF	0.049	0.020	0.057	0.021	-0.018	0.031
**Norm. absolute torque 75 ms**							
	PF	0.134	0.046	0.133	0.037	-0.053	0.054
	AZ	0.110	0.029	0.124	0.027	-0.043	0.071
	DF	0.123	0.049	0.131	0.044	-0.079	0.086
**Norm. absolute torque 100 ms**							
	PF	0.229	0.068	0.236	0.057	-0.093	0.111
	AZ	0.198	0.044	0.214	0.049	-0.068	0.101
	DF	0.210	0.064	0.226	0.058	-0.076	0.093
**Norm. absolute torque 125 ms**							
	PF	0.332	0.090	0.349	0.070	-0.111	0.145
	AZ	0.270	0.067	0.305	0.071	-0.094	0.158
	DF	0.286	0.080	0.309	0.071	-0.146	0.172
**Norm. absolute torque 150 ms**							
	PF	0.404	0.082	0.440	0.085	-0.097	0.177
	AZ	0.365	0.095	0.384	0.091	-0.037	0.098
	DF	0.373	0.093	0.386	0.087	-0.129	0.152
